# Molecular Control of Carpel Development in the Grass Family

**DOI:** 10.3389/fpls.2021.635500

**Published:** 2021-02-16

**Authors:** Chaoqun Shen, Gang Li, Ludovico Dreni, Dabing Zhang

**Affiliations:** ^1^Joint International Research Laboratory of Metabolic and Developmental Sciences, State Key Laboratory of Hybrid Rice, Jiangsu Collaborative Innovation Center of Regional Modern Agriculture & Environmental Protection, School of Life Sciences and Biotechnology, Shanghai Jiao Tong University, Shanghai, China; ^2^School of Agriculture, Food and Wine, University of Adelaide, Urrbrae, SA, Australia; ^3^Instituto de Biología Molecular y Celular de Plantas, Consejo Superior de Investigaciones Científicas-Universidad Politécnica de Valencia, Valencia, Spain

**Keywords:** carpel, carpel identity, meristem determinacy, plant hormones, miRNA, grass

## Abstract

Carpel is the ovule-bearing female reproductive organ of flowering plants and is required to ensure its protection, an efficient fertilization, and the development of diversified types of fruits, thereby it is a vital element of most food crops. The origin and morphological changes of the carpel are key to the evolution and adaption of angiosperms. Progresses have been made in elucidating the developmental mechanisms of carpel establishment in the model eudicot plant *Arabidopsis thaliana*, while little and fragmentary information is known in grasses, a family that includes many important crops such as rice (*Oryza sativa*), maize (*Zea mays*), barley (*Hordeum vulgare*), and wheat (*Triticum aestivum*). Here, we highlight recent advances in understanding the mechanisms underlying potential pathways of carpel development in grasses, including carpel identity determination, morphogenesis, and floral meristem determinacy. The known role of transcription factors, hormones, and miRNAs during grass carpel formation is summarized and compared with the extensively studied eudicot model plant *Arabidopsis*. The genetic and molecular aspects of carpel development that are conserved or diverged between grasses and eudicots are therefore discussed.

## Introduction

**FIGURE 1:**
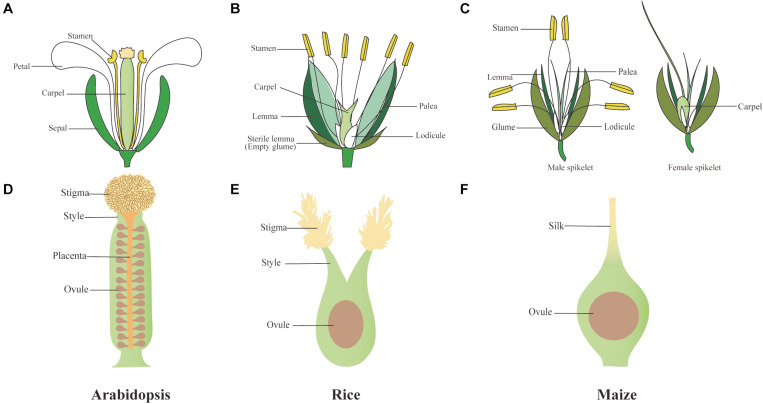
Schematic draws of *Arabidopsis*, rice, and maize florets and gynoecia. **(A–C)** Flower and spikelet structure of *Arabidopsis* **(A)**, rice **(B)**, and maize **(C)**. **(D)** The pistil of *Arabidopsis* can be divided into ovary, style, stigma, and the ovules derive from a placenta **(E)** Rice syncarpic ovary harbors two styles and feathery stigmas, **(F)** while maize has a higher extent of fusion into one silk. Like in all grasses, only one ovule is developed in the pistil of rice and maize.

Carpels are a major defining feature of angiosperms. This distinctive female reproductive structure occupies the center of the flower (Figure 1), encloses ovules, and greatly improves reproductive efficiency compared with gymnosperms, involving a more complex and diversified process of pollination. Among species, the female reproductive organ, gynoecium or pistil, may occur as single carpel, multiple independent carpels, or a fused syncarpic structure (Gomariz-Fernández et al., 2017). A basic organization plan composed of three distinct regions can be found across angiosperms carpels despite their huge morphological diversity: the basal ovary, a style, and an apical stigma (Figure 1) (Becker, 2020). Poaceae, the grass family, is one of the largest families of angiosperms; it includes many agriculturally important crops, such as domesticated rice (*Oryza sativa*), barley (*Hordeum vulgare*), wheat (*Triticum aestivum*), and maize (*Zea mays*). The spikelet is a basic structure unit of the inflorescence in all grass family, which in rice contains a fertile floret and two depressed sterile lemmas (also called empty glumes). Within each rice floret, there are two bract-like organs called lemma and palea that surround the inner floral organs, the lodicule, stamen, and carpel (Figure 1B). Maize has a similar flower structure with rice, although maize flowers undergo sex determination after floral organ initiation, with only stamens and carpels developing in tassel and ear spikelet, respectively (Figure 1C). As the innermost floral organ, rice, barley, and wheat pistils display a similar structure, which is derived from the early fusion of three carpel primordia, with two partially fused styles and two feathery stigmas covered with papillae cells where pollen is deposited (Figure 1E) (Kellogg, 2001; Rudall et al., 2005; Dreni et al., 2013). In maize, the single pistil is formed by three connate carpels; however, only the two lateral-abaxial carpels will develop to an elongated silk, whereas the growth of medial-adaxial carpel is inhibited before enveloping the single ovule (Figure 1F) (Strable and Vollbrecht, 2019). In comparison, the pistil of model eudicot *Arabidopsis thaliana* consists of two fused lateral carpels that bear submarginal ovules in the two locules (Figure 1D) (Becker, 2020).

During the past two decades, advances in ancestral state reconstruction, phylogenetics and developmental genetics have shed some light on carpel evolution. Depending on whether carpel is considered to be modified from male or female structures in their presumed gymnosperm-like ancestor, several potential hypotheses for the origin of the flower and its carpel exist. The Mostly Male Theory (Frohlich, 2003) postulates the first carpels are formed after the ectopic ovules generated on male microsporophylls. The Out-of-Male (Theissen et al., 2002) and its sister theory Out-of-Female (Theissen et al., 2002) hypothesis propose that the bisexuality of the flower is arisen by expression movements of the male (B-class genes) and female-promoting gene (B-sister genes), thus leaving female structures at the apex and male structures in basal region. Baum and Hileman (2007) proposed a central role of gene *LEAFY* (*LFY*) in the origin of the flower, which regulates C-function MADS-box genes in the flowering plants ancestor; these C-class genes then would have specified apical female reproductive structure, which later became carpels (Vialette-Guiraud and Scutt, 2009; Ferrándiz et al., 2010). Recently, the carpel of *Anaxagorea*, the most basal genus of primitive angiosperms Annonaceae (Magnoliales), is studied (Li et al., 2020). Supports are provided in this study for another carpel origin theory called the stachyosporous theory, which suggests that carpel originated from a compound shoot of the ovular axis and foliar appendage (Hickey and Taylor, 1996; Li et al., 2020). Besides these theories, a basic ancestral angiosperm carpel developmental networks has been proposed (Becker, 2020), involving *STYLISH* (*STY*)-, *SPATULA* (*SPT*)-, *ETTIN* (*ETT*)-, and *CRABS CLAW* (*CRC*)-like genes in basal/apical and adaxial/abaxial polarity specification and growth, and *HECATE* (*HEC*) homologs regulating only longitudinal growth. Homologs of *NGATHA* (*NGA*) and *STY*/*SHORT INTERNODES* (*SHI*)*/SHI RELATED SEQUENCE* (*SRS*) may have already contributed in regulating stigmatic-like tissue development in the carpel of the hypothetic aMRCA (angiosperm most recent common ancestor), while it probably lacked a style (Fourquin et al., 2005; Endress and Doyle, 2009, 2015; Soltis and Soltis, 2016; Becker, 2020).

To date, most data on the gene regulatory networks involved in tissue differentiation and patterning during gynoecium morphogenesis processes are still derived from *A. thaliana*. The major roles of phytohormones, many transcription factors (TFs), and coregulators have been identified in specifying tissue orientations and gynoecium formation, which have been extensively reviewed in *Arabidopsis* (Reyes-Olalde et al., 2013; Pfannebecker et al., 2017a, b; Reyes-Olalde and de Folter, 2019; Simonini and Østergaard, 2019; Zúñiga-Mayo et al., 2019). Molecular genetic studies have uncovered developmental mechanisms of carpel establishment in two grass model species, rice and maize. In view of a similar carpel development pattern in grasses, these studies will facilitate related research in the future, including barley, wheat, and other cereals. Here, we update focused studies mainly in rice and maize, on the regulatory mechanisms, networks, and pathways underlying the establishment and regulation of carpel identity, morphogenesis and development, and their intrinsic connection with floral meristem determinacy (FMD).

## Carpel Identity Determination

Like any floral organ, carpels initiate from meristem primordia, which are small domes of undifferentiated cells, and then develop their specific properties and structure through a process called morphogenesis. There are some key regulators identified in grass carpel identity determination whose functions are either conserved or diverged versus *Arabidopsis*.

**FIGURE 2:**
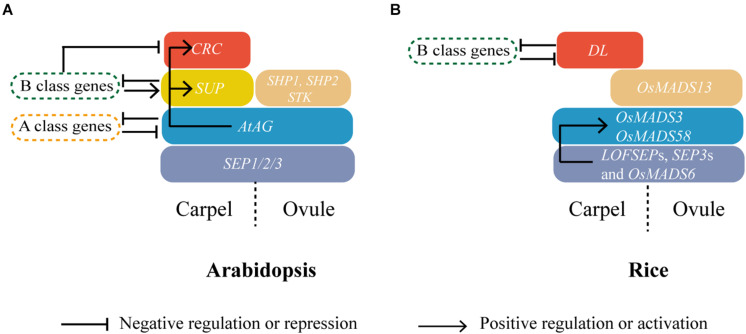
Carpel specification in dicot and monocot model plants *Arabidopsis* and rice. The expression domains of C-, D-, and E-class genes; *SUP*; and *CRC*, *DL* in carpel and ovule of *Arabidopsis* **(A)** and rice **(B)** are shown with blue, brown, purple, yellow, and red colors, respectively. Genetic interactions within these carpel identity regulators and other homeotic genes are also indicated. Direct regulations have been shown in *Arabidopsis*, whereas in rice a direct binding of OsMADS6 has only been shown to the second intron of *OsMADS58* (Li et al., 2011a).

Genetic studies based on *Arabidopsis* and *Antirrhinum majus* led to the formulation of the ABC model of flower development and floral organ identity (Coen and Meyerowitz, 1991; Weigel and Meyerowitz, 1994) that predicts the combinatorial activity of three classes of genes as follows: A-class genes specify sepal identity in the first whorl, A- and B-class genes specify petal identity in the second whorl, and B- and C-class genes are necessary for stamen identity in the third whorl, whereas C-class genes alone are required for carpel identity and FMD in the fourth and last whorl in the center of the flower. Later studies in *Petunia hybrida* and *Arabidopsis*, proposed that a D class of genes is specifically required for ovule development and an E class of genes is essential for the function of all the other four classes, which led to an updated ABCDE model (Colombo et al., 1995; Pelaz et al., 2000, 2001; Honma and Goto, 2001; Favaro et al., 2003; Pinyopich et al., 2003). Except for the *Arabidopsis* A class *APETALA2* (*AP2*) gene, all the ABCDE genes encode for MIKC-type MADS-box TFs working in tetrameric complexes (Riechmann et al., 1996; Theißen, 2001; Paøenicová et al., 2003; Theißen et al., 2016). While the conservation of the model for the outer whorls is amply debated (Litt, 2007; Causier et al., 2010), the C and D functions are widely conserved in carpel and ovule identity among angiosperms, which largely depends on MADS-box genes from the *AGAMOUS* subfamily (Figure 2A) (Dreni and Kater, 2014). In addition, complexes formed by their encoded proteins with E class proteins that are encoded by *SEPALLATA* subfamily MADS-box genes seem also conserved (Figure 2A) (Dreni and Kater, 2014).

The *AGAMOUS* subfamily is named after its founding member gene, *Arabidopsis AGAMOUS* (*AtAG*). *AtAG*, as a C-class gene, is the master regulator of stamen and carpel formation (Bowman et al., 1989, 1991; Yanofsky et al., 1990; Lohmann and Weigel, 2002). FMD and reproductive organ identity are completely lost in the *ag* mutant, leading to a continuous repetition of sepal–petal–petal whorls forming indeterminate flowers (Bowman et al., 1989, 1991).

### The Role of *AGAMOUS* Genes in Carpel Identity Determination in Grasses

Rice has two *AtAG* orthologs, *OsMADS3* and *OsMADS58*, which share a very similar expression profile; both express exclusively in whorl 3 (stamen) and whorl 4 (carpel), although *OsMADS58* is expressed more uniformly throughout the floral meristem (FM) (Yamaguchi et al., 2006; Dreni et al., 2011). In rice *osmads3-4* (Hu et al., 2011) and *osmads3-fe1* (Yasui et al., 2017) single mutants, most flowers display a normal single pistil, whereas in the reported null allele *osmads3-3* (Yamaguchi et al., 2006), 84% of the flowers showed increased carpel numbers and multiple stigmas, and enlarged or elongated ovules are also formed. Interestingly, this loss-of-function allele is generated by a T-DNA insertion in the large second intron (Yamaguchi et al., 2006), suggesting evolutionary conserved importance of this region in promoting the correct expression of *AG-*like genes, in agreement with studies conducted in *Arabidopsis* and *Antirrhinum* (Sieburth and Meyerowitz, 1997; Causier et al., 2009). Despite the similar expression patterns between *OsMADS3* and *OsMADS58*, *osmads58* single mutants display normal flowers; only 5% of those have bifurcated stigmas (Dreni et al., 2011). In both *osmads3-3 osmads58* and *osmads3-4 osmads58* double mutants, ectopic lodicules and green palea/carpel-like organs repeatedly replace stamen and carpel, respectively. With similar abaxial surface, Dreni et al. (2011) interpreted the organs replacing carpels in *osmads3-3 osmads58* as palea-like, having a similar structure, and a body and two margin regions, which are characteristics of palea (Prasad et al., 2001; Prasad and Vijayraghavan, 2003). Surprisingly, this clear morphological transformation does not match with an equally obvious change in the expression of the only known putative marker genes: although the glume marker *G1/ELE* (*long sterile lemma/elongated empty glume*) is activated in the indeterminate FM of *osmads3-3 osmads58*, it does not mark clearly the emerging palea-like primordium that, in addition, continues to express *DROOPING LEAF* (*DL*), a gene required for carpel identity specification, like in the wild-type ovary wall (Dreni et al., 2011).

A residual carpel identity was discovered in the adaxial epidermis of the *osmads3-3 osmads58* aberrant gynoecium, conferred by another *AGAMOUS* subfamily member, the D class ovule identity gene *OsMADS13* (Dreni et al., 2007). *OsMADS13* is expressed in the adaxial epidermis of the ovary wall of wild-type plants, and this pattern is maintained also in the adaxial epidermis of the palea/carpel-like organ of *osmads3-3 osmads58* flower. Indeed, the residual carpel identity is completely eliminated by generating *osmads3-3 osmads13 osmads58* triple mutants, whose palea-like structures grow even more and present an adaxial surface completely equivalent to a palea. Yet, *DL* expression remains strong in these organs (Dreni et al., 2011).

Recently, the identity of green organs replacing the carpel in the *osmads3 osmads58* double mutants has been questioned by Sugiyama et al. (2019), who generated a new double-mutant *osmads3-fe1 osmads58-7* for their analysis. Different from *osmads3-3 osmads58* in which *OsMADS58* is mutated by a *dSpm* transposon insertion in the second intron (Dreni et al., 2011), in *osmads3-fe1 osmads58-7*, the ∼600-bp promoter region and the first three exons of *OsMADS3* are deleted, and there is a frame-shift mutation in exon 3 of *OsMADS58* (Yasui et al., 2017; Sugiyama et al., 2019). Despite revealing that the green ectopic organs in the fourth whorl express the A-class and palea identity gene *OsMADS15*, which is also in agreement with the ABC model, Sugiyama et al. (2019) claim that the abaxial surface still retains features of the ovary cells. Based on this, they reinterpreted the fourth whorl organs of *osmads3-fe1 osmads58-7* as carpel-like and proposed that the persisting expression of *DL* and *OsMADS13* is supportive of carpel-like identity, while the palea-like structure is conferred by ectopic expression of the A class gene *OsMADS15*. In conclusion, Sugiyama et al. (2019) suggest that rice C-class genes are not essential for carpel identity. Future works will be needed to clarify this essential question.

Another recent study has suggested an important functional role for the conserved amino acidic residue 109 of AG proteins in grasses (Dreni et al., 2020). It is shown that an alternative splicing occurs on the *OsMADS3* transcript of *O. sativa*, producing two variants of OsMADS3 that differ just for the presence or absence of a conserved serine residue, S109. Interestingly, this splicing variant is conserved and specific between grass families. Only the eudicot-like OsMADS3 isoform, lacking S109, is able to specify stamens and carpels in *Arabidopsis*, as revealed by functional complementation assay expressing the two rice isoforms in the *Arabidopsis ag* mutant (Dreni et al., 2020). It is further proposed that the different ability between OsMADS3^+S109^ and OsMADS3 in complementing the *ag* phenotype might be partially explained by their different interactions with diverse SEPALLATA-like proteins (Dreni et al., 2020). Therefore, the two isoforms of OsMADS3-like proteins might have partially distinct function in grasses.

In maize, there are three *AG* lineage genes, with *ZAG1* ortholog to *OsMADS58* and *ZMM2* and *ZMM23* orthologs to *OsMADS3* (Schmidt et al., 1993; Theißen et al., 1995; Münster et al., 2002). Unlike rice, maize *AG* orthologs appear to express in spatially distinct domains, as *ZAG1* and *ZMM2* transcript levels are higher in carpel and stamen, respectively. A mutation in *ZAG1* results in unfused carpels in female flowers, but the carpel identity is not affected (Mena et al., 1996). Functional analysis of *ZMM2* and *ZMM23* has not been reported yet.

The putative C class genes of wheat, *WAG1* (wheat *AGAMOUS1*) and *WAG2*, have been identified as *OsMADS58* and *OsMADS3* orthologs, respectively (Meguro et al., 2003; Zhao et al., 2006; Wei et al., 2011). In early floral organ initiation stages, the transcription level of the two *WAG* genes is low, but transcripts are detected from booting to heading stages, and *in situ* hybridization analysis shows *WAG* expression in stamen, carpel, and ovule (Meguro et al., 2003; Yamada et al., 2009). Besides, ectopic expression of *WAG1* and *WAG2* can induce pistilloid ectopic stamens in an alloplasmic wheat line (Yamada et al., 2009). Collectively, these data suggest a partially conserved function of wheat *AG* orthologs in floral organ development, while their lower expressions detected in early stages may also indicate a minor function in carpel differentiation (Callens et al., 2018).

### The Role of *DL* in Carpel Identity Determination in Grasses

The *Arabidopsis CRABS CLAW* (*CRC*), a YABBY family TF, is required for maintaining the proper growth of the gynoecium (Alvarez and Smyth, 1999). *CRC* is expressed throughout nectary development (Baum et al., 2001). In the gynoecium, *CRC* is first expressed in the pistil primordium, and subsequently, only in the abaxial epidermis of the carpel and in four internal domains adjacent to the cells that will form the placenta, then its expression disappears after the initiation of ovule primordia (Bowman and Smyth, 1999; Colombo et al., 2010). In the *crc* mutant, style growth is reduced, resulting in incomplete carpel fusion (Alvarez and Smyth, 1999). It is also reported that the *Arabidopsis CRC*, a direct target of *AtAG* (Gómez-Mena et al., 2005; Ó’Maoiléidigh et al., 2013), acts in mediating the transition from FM termination to gynoecium development (Figure 2A) (Yamaguchi et al., 2017). The rice *CRC* ortholog *DL* is proposed to be required for carpel identity, because in strong *dl* mutant alleles, carpels undergo homeotic transformation to stamens (Yamaguchi et al., 2004). In rice, *DL* expression can be first detected in a few cells at the lemma side of FM, and later during gynoecium development, *DL* expression starts in the carpel anlagen, shortly before carpel primordia begin to form. After carpel initiation, *DL* is expressed specifically and uniformly in carpel primordia, whereas no expression is detected in the FM or ovule primordium, which differs from both *CRC* and the typical *AG*-like C-function genes (Yamaguchi et al., 2004). *DL* seems to act mutually and antagonistically to the class B gene *SUPERWOMAN1* (*SPW1*)/*OsMADS16* to control carpel identity in rice (Figure 2B), as ectopic stamens replace the gynoecium in *dl*, whereas homeotic transformation of stamens into carpels occurs in *spw1-1*. Moreover, the *SPW1* and *DL* transcripts can be detected in the ectopic stamens in the fourth whorl of *dl* and in the ectopic carpels of *spw1-1* mutants, respectively (Nagasawa et al., 2003; Yamaguchi et al., 2004). In *Arabidopsis*, *CRC* expression is also found to be expanded in B class mutants *ap3* and *pi* (Bowman and Smyth, 1999; Wuest et al., 2012). However, besides the homeotic transformation, unidentifiable floral organs are indeterminately produced in whorls 3 and 4 of the rice *spw1-1 dl* double mutants (Nagasawa et al., 2003; Sugiyama et al., 2019). These findings suggest that *DL*, besides regulating the boundary between whorls 3 and 4, may play an active role in carpel identity determination (Figure 2B). In comparison, *Arabidopsis CRC* seems to have a more specific function in regulating some aspects of carpel development and is not required to exclude the B class genes from the gynoecium, a function played instead by the *Arabidopsis* C2H2 zinc finger TF encoded by *SUPERMAN* (*SUP*), which is also positively regulated by B class genes themselves in the early stages (Figure 2A) (Schultz et al., 1991; Bowman et al., 1992; Bowman and Smyth, 1999; Sakai et al., 2000).

In wheat, the *DL* ortholog has been identified by homology screening (Ishikawa et al., 2009). *TaDL* is also expressed in the wheat pistil, as well as in the ectopic pistil-like primordia replacing stamens in a pistillody line (Hama et al., 2004; Ishikawa et al., 2009). Moreover, the wheat B class MADS-box genes are expressed as expected in the wild-type stamen primordia, but similar to the case in rice, transcripts are not detected in those ectopic carpels in the pistillody line (Hama et al., 2004). Taken together, these facts suggest that a mutual repression between *DL* and B class genes is conserved in grasses, although this is not yet clear whether it is direct or not.

### Models for Grass Carpel Identity Regulation

In *Arabidopsis*, based on the ABCDE model, carpel identity is specified by the C function gene *AtAG* and E function genes *SEPALLATA1/2/3* with the mutual repression between the A- and C-function genes in their respective domains (Figure 2A) (Pelaz et al., 2000, 2001; Causier et al., 2010). In contrast, the carpel identity regulation model in rice is more controversial, based on the different alleles studied, and/or diverse interpretations on *osmads3*, *osmads58*, and *dl* mutants, currently two different theories exist. Dreni et al. (2011) proposed that *OsMADS3* and *OsMADS58* still have conserved class C protein functions and redundantly regulate carpel identity, whereas Dreni et al. (2011) implicated *DL* as a fourth-whorl marker and negative regulator of class B genes, without a direct carpel identity specification function, as *DL* is unable to specify carpel identity in the absence of *OsMADS3* and *OsMADS58*. In contrast, Sugiyama et al. (2019) claimed a dispensable role of *OsMADS3* and *OsMADS58* for carpel specification, which would be the first known case in angiosperms, and that *DL* is the key determinant of carpel identity. However, there is no functional carpel in either *spw1-1 dl* or *osmads3 osmads58* double mutants, suggesting that *AG*-like genes or *DL* alone is not sufficient to induce carpel formation. Thus, it is more plausible to conclude that *DL*, *OsMADS3*, and *OsMADS58* are all necessary for carpel identity specification and morphogenesis (Figure 2B). Besides the above three regulators, *OsMADS13*, the D function gene of rice (Dreni et al., 2007), also plays a role in carpel establishment, although limited to its adaxial side, as we described above (Figure 2B). This is in contrast with *Arabidopsis*, where class C and D genes redundantly confer ovule identity, but carpel identity is determined exclusively by C function genes (Figure 2A) (Pinyopich et al., 2003).

Grasses have diverse *SEP*-like genes that can be divided into two subclades, in rice the three genes *OsMADS1*, *OsMADS5*, and *OsMADS34* belong to the *LOFSEP* clade, whereas the *SEP3* clade consists of the other two members *OsMADS7/45* and *OsMADS8/24* (Malcomber and Kellogg, 2005; Zahn et al., 2005; Arora et al., 2007). Similar with *Arabidopsis*, higher-order knockdown and mutants of rice *SEP* genes cause the homeotic transformation of floral organs to leaf-like structures, and the rice *SEP* members also can form heterodimers with C- and D-class genes, suggesting a conserved pivotal role of *SEP* genes in carpel identity specification (Pelaz et al., 2001; Cui et al., 2010; Wu et al., 2017). However, functional diversification of rice *SEP* genes also exists compared with *Arabidopsis*, as inhibition of *OsMADS1* or *OsMADS34* alone, and *OsMADS7* and *OsMADS8* together are sufficient to cause severe phenotypes including leafy lemma and palea, homeotic transformation of lodicules, fewer stamens, increased carpels, and flowering time alterations (Cui et al., 2010; Gao et al., 2010). Carpels are either increased or completely missing in several combinations of *lofsep* mutants (Wu et al., 2017). The simultaneous knockdown of *OsMADS7* and *OsMADS8* causes different degrees of carpel fusion defects, also affecting the FMD with additional reproductive organ-like structures initiated inside the mutant carpels (Cui et al., 2010).

Recently, some potential regulations between rice *SEP* genes and *AG* genes have been discovered. As the expression of *OsMADS3* and *OsMADS58* significantly decreased in *osmads1-z* and *lofsep* double and triple mutants, it is proposed that rice *LOFSEP* members may act as upstream activators of C-class genes to regulate inner floral organs (Figure 2B) (Wu et al., 2017). Indeed, consistent with the delayed transition of the spikelet meristem to FM, the initiation of *OsMADS3* expression is delayed, and *OsMADS58* expression is missed from the FM in *osmads1-z* mutants (Hu et al., 2015). Moreover, another recent study (Osnato et al., 2020) has identified a substantial overlap with DEGs from the transcriptome data of *osmads1* (Khanday et al., 2013; Hu et al., 2015) and *osmads13* mutants, suggesting the two genes may regulate common downstream targets but in an antagonistic manner. The authors also proposed that *OsMADS13* may act as a repressor in the carpel initiation pathway in the ovule domain (Osnato et al., 2020).

In short, these findings suggest that *OsMADS3*, *OsMADS58*, *DL*, *OsMADS13*, and *SEP* genes in rice have both conserved and diverse functions in carpel identity determination and morphogenesis, compared with *Arabidopsis*. These functional diversifications may play a pivotal role in establishing rice carpel morphology, and it is highly likely that similar cases also exist in other grass plants such as maize, barley, and wheat.

## Floral Meristem Determinacy in Carpel Development

Unlike indeterminate meristems such as the shoot apical meristem and inflorescence meristem in *Arabidopsis* and some grass species, the FM is determinate, as the stem cells in FM will be consumed by the final floral organ initiated from it. The pattern of FM termination is diverse among angiosperm species. In wild-type rice flower, the carpel primordia bulge first arises at the lemma side of the FM, then at the flank of the meristem; the other carpel primordia develop toward the opposite side to enclose the meristem. The FM remains morphologically undifferentiated and later develops into the ovule (Itoh et al., 2005). But in *Arabidopsis*, the stem cell maintenance ceases when carpel primordium starts to be initiated, and ovules differentiate from the placenta after establishment of the carpel (Cucinotta et al., 2014). Thus, the central region of the FM is consumed by the ovule or carpel/placenta primordium in rice and in *Arabidopsis*, respectively.

### Interactions of the *CLV-WUS* Pathway and *APO1* With Carpel Identity Genes

Interactions between floral homeotic genes and the well-characterized *CLAVATA* (*CLV*)-*WUSCHEL* (*WUS*) negative feedback pathway, which coordinates stem cell proliferation with differentiation in the meristem, are part of FMD regulation (Brand et al., 2000; Schoof et al., 2000; Ikeda et al., 2009; Pautler et al., 2013). In *Arabidopsis*, the stem cell-promoting gene *WUS* is able to induce *AtAG* expression, whereas *AtAG* in turn can switch off *WUS* right after carpel primordia emergence, through several molecular mechanisms, to trigger FM termination in the center of the flower bud (reviewed by Shang et al., 2019). Mutations of the receptor kinase-encoding gene *CLV1*, another component of the CLV-WUS pathway, can trigger defects in *AtAG* expression (Clark et al., 1993). Grass class C genes also play an important role in FMD. Like the *Arabidopsis ag* mutant, *osmads3 osmads58* double mutants show a dramatic loss of FMD, as an indefinite number of ectopic lodicules and green palea/carpel-like organs are repeatedly formed (Dreni et al., 2011; Sugiyama et al., 2019), and the FM marker gene *OSH1* (*O. sativa homeobox1*) expression remains even in mature flowers (Sugiyama et al., 2019). Loss of function in maize *ZAG1* also results in the partial loss of FMD in female flowers, showing a reiteration of carpels (Mena et al., 1996). The rice *FON1* and *FON2*/*FON4* genes are identified as orthologs of *CLV1* and *CLV3*, respectively, an alteration in floral organ number and increased FM size have been observed in *fon1-1* (Moon et al., 2006) and *fon4-1* (Chu et al., 2006) mutants, suggesting that the function of the CLV3-CLV1 ligand–receptor system is conserved. The organ number is more severely affected in the inner whorls in those mutants, and nearly all *fon4-1* and *fon4-2* flowers have from 2 to 10 carpels (Chu et al., 2006). Both *fon4-2 osmads3-4* and *fon4-1 osmads58* double mutants display enhanced defects of FMD with more carpel-like structures than either single mutants. The *fon4-2 osmads3-4* double mutant has a dramatically enlarged FM size and larger *OSH1* expression region, compared with wild-type plants, whereas the expression of *OsMADS3* and *OsMADS58* in *fon4-2* mirrored that in the wild type. In conclusion, *FON4* synergistically interacts with C-class genes in rice FMD (Xu et al., 2017; Yasui et al., 2017).

As described above, ovule is the final organ differentiated from FM in grasses. Mutation in the ovule identity specification gene *OsMADS13* also results in partial loss of FMD. Reiteration of carpels and prolonged expression of *OSH1* have been observed in *osmads13* mutants (Dreni et al., 2007; Yamaki et al., 2011). The number of carpelloid structures in the *fon4-1 osmads13-3* double mutant is greatly increased in comparison with the single mutants, and the expression domain of *OSH1* is wider. Besides, the expression level of *OsMADS13* is increased and more widely detectable in the gynoecium of *fon4-2* mutant (Xu et al., 2017). Thus, *FON4* may function to repress *OsMADS13* expression in the innermost whorl, or the increased expression of *OsMADS13* may act as a molecular response to alleviate the expansion of the meristem in *fon4-2* (Xu et al., 2017).

In *Arabidopsis*, *LFY* and *UNUSUAL FLORAL ORGAN* (*UFO*) are reported to be key factors in enhancing the floral fate of lateral meristems and regulating the ABC floral organ identity genes (Weigel et al., 1992; Lee et al., 1997; Parcy et al., 1998; Chae et al., 2008). In rice, *ABERRANT PANICLE ORGANIZATION 2* (*APO2*, ortholog of *LFY*) and *ABERRANT PANICLE ORGANIZATION 1* (*APO1*, ortholog of *UFO*) act cooperatively in controlling inflorescence and flower development. Both *apo1* and *apo2* mutants display partial loss of FMD in whorl 4 with indeterminate carpel formation, which is even more severe in the *apo1-1apo2-1* double mutant (Ikeda et al., 2005; Ikeda-Kawakatsu et al., 2012). Furthermore, the expression of C class gene *OsMADS3* is down-regulated in *apo1*. Considering that the *osmads3* loss-of-function mutants form *apo1*-like flowers, it is reasonable to propose a positive regulation of the C class gene *OsMADS3* by *APO1*, but not of B class genes as in *Arabidopsis* (Lohmann and Weigel, 2002; Ikeda et al., 2005).

### Interactions Within Floral Homeotic Genes

Interactions within floral homeotic genes are also important in determining FM fate. Similar to *Arabidopsis* (Mizukami and Ma, 1995), FMD in rice seems to be sensitive to the amount of AG-like protein(s), as all the three double mutants (*osmads3-3 osmads58*, os*mads3-3 osmads13*, and *osmads13 osmads58*) result in an enhanced indeterminacy in the fourth whorl (Dreni et al., 2011). In addition, *OsMADS13* and *OsMADS3* do not regulate each other’s transcription, suggesting a synergistic role of these two genes (Li et al., 2011b).

During FM termination in *Arabidopsis*, *AtAG* represses *WUS* in part by directly activating three key target genes, the C2H2 zinc finger genes *KNUCKLES* (*KNU*) and *SUP*, and the YABBY gene *CRC* (Alvarez and Smyth, 1999; Payne et al., 2004; Gómez-Mena et al., 2005; Lee et al., 2005; Ó’Maoiléidigh et al., 2013). *SUP* and *CRC* contribute to FMD non-cell autonomously, at least in part by fine tuning auxin homeostasis (Yamaguchi et al., 2017; Lee et al., 2019).

**FIGURE 3:**
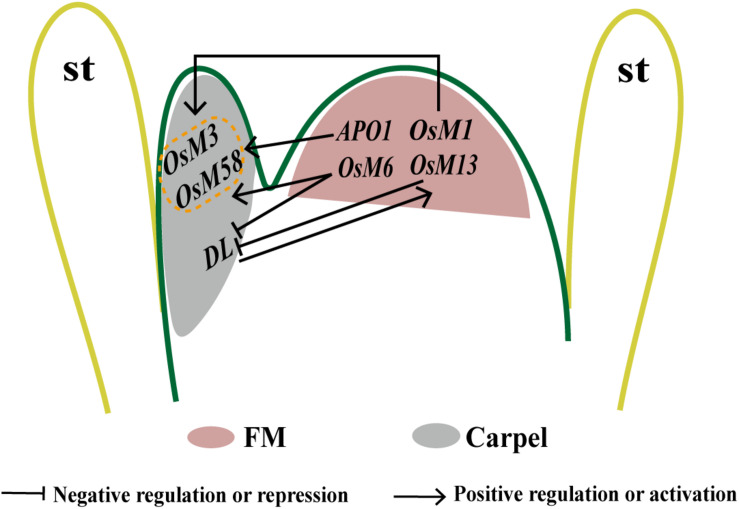
Proposed model to illustrate the genetic interaction between rice floral homeotic genes in FMD in whorl 4. *OsMADS3* and *OsMADS58* are possibly activated by E-function genes *OsMADS1* and *OsMADS6* and also *APO1*, the expression of *DL* may be repressed by *OsMADS6* and *OsMADS13*. In turn, *DL* may indirectly regulate *OsMADS13* in a positive way. The onset of *OsMADS13* expression in the final stage of the FM leads its transition to ovule primordium.

The rice ortholog of *CRC* is *DL*, which also functions as carpel identity regulator and, non-cell autonomously, in FMD (Yamaguchi et al., 2004; Sugiyama et al., 2019). In rice *dl* mutant, ectopic stamens are variable in number (Yamaguchi et al., 2004). The *osmads3-4 dl* double mutants display a severe loss of FMD and produced extra whorls of lodicule-like organs in the floral center (Li et al., 2011b). However, the expression patterns of *OsMADS3* and *DL* are not affected by each other; thus, *OsMADS3* and *DL* may terminate the FM in a synergistic way. On the contrary, *osmads13-3 dl* flowers exhibited the similar defects to those of *dl*, and the transcripts of *OsMADS13* is undetectable in *dl* flowers, suggesting that *DL* and *OsMADS13* may function in the same pathway to specify the identity of carpel/ovule and FMD (Figure 3) (Li et al., 2011b). In *Arabidopsis*, *CRC* is a direct target of both B class genes and of the C class gene *AtAG*, which act as repressors and as activators, respectively (Bowman and Smyth, 1999; Gómez-Mena et al., 2005; Wuest et al., 2012; Ó’Maoiléidigh et al., 2013). In rice, *DL* expression also expands outward in the third whorl in B class mutants (Yamaguchi et al., 2004), but it does not disappear in the palea-like organs replacing the pistil in *osmads3 osmads58* double and *osmads3 osmads13 osmads58* triple mutants, suggesting that its expression is independent by C class genes (Dreni et al., 2011). In *Arabidopsis*, *CRC* genetically interacts in the fourth whorl with another FMD regulator, *SUP*, and common downstream genes such as cytokinin- and auxin-related genes are found between these two TFs (Prunet et al., 2017; Lee et al., 2019). As rice *SUP* counterparts have not been studied yet, it will be interesting to assess their function in rice FMD and carpel development.

Recently, the maize *CRC* and *DL* co-orthologs *DRL1* and *DRL2* have been also reported to regulate FM activity and impose FMD from lateral floral organs in a non-cell-autonomous way (Strable and Vollbrecht, 2019). The *drl1* mutant ears are sterile, carpel walls remain unfused with underdeveloped silks, and an expanded nucellus-like structure is exposed in the center. The medial-adaxial carpel (determinate carpel) is greatly reduced in both *drl2* and *drl1 drl2* double mutants, and multiple lateral-abaxial carpels (indeterminate carpels) have been observed in the double mutants, indicating a prolonged FM activity (Strable and Vollbrecht, 2019). Genetic interaction and expression analyses using gene regulatory network inference suggest that *DRL1* and *ZAG1* target each other, and there is a common set of downstream genes involved in floral patterning and FMD (Strable and Vollbrecht, 2019).

As mentioned previously, physical and genetic interactions between *OsMADS1* and the two rice C-class genes are essential for the regulation of carpel identity specification and FM activity (Hu et al., 2015); 74.67% of *osmads1-z osmads3-4* flowers lack inner floral organs, but only have extra glume-like structures, and *OSH1* expression is lost at a later stage in this double mutant, suggesting a function of *OsMADS1* and *OsMADS3* together in repressing FM activity (Figure 3) (Hu et al., 2015). As another MADS-box gene with E-class functions in rice, genetic analysis reveals a key role of the *AGL6* subfamily member *MOSAIC FLORAL ORGAN 1 (MFO1)*/*OsMADS6* in early flower development (Li et al., 2011a). In the flower of *mfo1* mutants, the FMD is lost, and extra carpels or spikelets developed in *mfo1* florets (Ohmori et al., 2009). A complex regulation network also exists within *OsMADS6* and other carpel regulators. All flowers in *osmads6 dl* double mutant display an inflorescence-like structure in whorl 4, and ectopic expression of *DL* is present in the altered palea organ and ectopic carpels or abnormal ovules in *osmads6-1*. Taken together, it is proposed that *OsMADS6* may repress the expression of *DL*, whereas *DL* does not affect *OsMADS6* expression (Figure 3) (Li et al., 2011a).

Moreover, *OsMADS6* also interacts genetically with *OsMADS13*, *OsMADS3*, and *OsMADS58*. The flowers of *osmads6-1 osmads13-3* display more severe defects in carpel/ovule development and FMD than single mutants (Li et al., 2011a), its floral axis developing higher-order carpel-like organs expressing *DL*. There is no obvious regulation between *OsMADS6* and *OsMADS13* at the transcriptional level, although they have partial functional redundancy in carpel/ovule identity specification and FMD termination. Defects are intensified with even more carpels in the floral center of the *osmads6-1 osmads3-4* and *osmads6-1 osmads58* double mutants, and an inflorescence-like structure is produced in *osmads6-1 osmads3-4* flowers (Li et al., 2011a). In *osmads6-1*, the expression of *OsMADS3* seems much weaker and delayed, and expression of *OsMADS58* is reduced at the early stages. Furthermore, *OsMADS6* may regulate *OsMADS58* expression directly through binding to a CArG element in its second intron (Li et al., 2011a), suggesting that *OsMADS6* may promote C-class genes expression during early rice flower development (Figure 3).

Generally, FM activity is terminated after the formation of a fixed number and pattern of floral organs. In *Arabidopsis*, it has been shown that *AtAG* is a key regulator in abolishing FM by switching off *WUS* (Lohmann et al., 2001; Liu et al., 2011; Sun et al., 2019). In rice, interactions between *FON4*, *APO1*, and C- and D-function genes are shown to act in FMD. Also, the interactions between floral homeotic genes *DL*, *OsMADS3*, *OsMADS58*, *OsMADS13*, and E-function genes *OsMADS1* and *OsMADS6* suggest a regulatory module that fine-tunes floret patterning and FMD in rice (Figure 3) (Ohmori et al., 2009; Dreni et al., 2011; Li et al., 2011a,b; Hu et al., 2015; Strable and Vollbrecht, 2019).

## The Role of Plant Hormones in Carpel Development

Carpel is a relatively complex biological structure among the plant organs; it requires several tissues to acquire specific identities in the specific time and places. Studies in *Arabidopsis* have shown that gynoecium formation depends on precise hormonal and genetic interactions to produce this complex tissue organization (Marsch-Martínez et al., 2012; Marsch-Martínez and de Folter, 2016; Moubayidin and Østergaard, 2017). Several recent reviews well-summarized the role of plant hormones such as auxin, cytokinin, and their crosstalk and the genes that connect these hormonal pathways during gynoecium development processes such as apical-basal and mediolateral polarity establishment, identity specification, symmetry establishment, and patterning (Moubayidin and Østergaard, 2017; Deb et al., 2018; Zúñiga-Mayo et al., 2019). The developmental control by plant hormone in the carpel margin meristem, which is the inner tissues possessing meristematic characteristics in the *Arabidopsis* gynoecium, is also addressed in several other reviews (Reyes-Olalde et al., 2013; Reyes-Olalde and de Folter, 2019).

### Auxin

Auxin is an essential hormone for almost all developmental processes in plants; experimental and modeling approaches have demonstrated that organ initiation depends on a threshold concentration of auxin (Reinhardt et al., 2003). Disruption of the *Arabidopsis* and maize protein kinase *PINOID* (*PID*), which is involved in polar auxin transport, causes severe defects in flower initiation. *Arabidopsis pid* and maize ortholog *bif2* (*BARREN INFLORESCENCE2*) mutants display defects in flower initiation that results in a pin-like inflorescence (Bennett et al., 1995; McSteen et al., 2007). Surprisingly, despite the fact that *OsPID* expresses throughout the panicle development as well as in pistil, none of the *ospid* mutants develops pin-like inflorescences. Instead, stigma development is completely eliminated in *ospid* mutants and normal ovules with eight-nucleate embryo sac failed to develop in most spikelets (He et al., 2019; Xu et al., 2019), whereas the overexpression of *OsPID* led to overproliferation of stigmas (Morita and Kyozuka, 2007). These observations suggest a key role of *OsPID* for stigma development and ovule initiation.

In *ospid* mutant, the expression of most auxin response factor genes is shown to be downregulated, and *OsETTIN1*, *OsETTIN2*, and *OsMONOPTEROS (OsMP)* lost their original spatiotemporal expression pattern during pistil development (Xu et al., 2019). As *ETTIN* and *MP* play pivotal roles in FM maintenance, *OSH1* transcription signal is weaker, and *FON4* transcription level is higher in *ospid*, it is proposed that *OsPID* may regulate stigma and ovule initiation by maintaining stem cell identity through auxin signaling (Xu et al., 2019). A recent study shows that *OsPID* also interacts physically with *OsMADS16* (Wu et al., 2020); thus, *OsPID* may be involved in carpel development by interacting with floral homeotic genes. Indeed, another auxin-responsive gene *OsMGH3* (*OsMADS1 regulated GH3 domain-encoding gene*) has been reported as a common downstream target of *OsMADS1* and *OsMADS6* (Prasad et al., 2005; Zhang et al., 2010). *GH3* control cellular bioactive auxin by inactivating excess auxin as conjugates of amino acids and sugars to maintain auxin homeostasis (Woodward and Bartel, 2005). *OsMGH3* knockdown plants display partial overlapped phenotypes with *osmads1* and *osmads6*; carpel development and pollen viability are affected by reduced fertility in dsRNAi*OsMGH3* mutant plants (Yadav et al., 2011). Besides, 10%–20% of *OsMGH3* knockdown transgenic flowers show enlarged carpels, which protrudes out of the flower before anthesis (Yadav et al., 2011). Such enlarged carpel phenotypes are similar to *Arabidopsis ettin* and *arf2* that are mutants in auxin response factors, suggesting the key role of auxin-responsive genes regulation in plant carpel development (Sessions et al., 1997; Okushima et al., 2005).

### Cytokinin

Cytokinin is a key regulator of meristem size and activity by controlling cell division and differentiation and thus influences numerous developmental programs in plants. The rice *LONELY GUY* (*LOG*) gene encodes a cytokinin-activating enzyme functioning in the final step of bioactive cytokinin synthesis. The mRNA of *LOG*, which is required to maintain meristem activity, is specifically detected in shoot meristem tips, and its loss-of-function mutant displays premature termination of the shoot meristem (Kurakawa et al., 2007). In the *log-3* weak allele, 65.3% of spikelets form a slender pistil lacking an ovule, whereas 15.8% form no pistil. The FM volume in *log-3* mutant is normally maintained until carpel protrusion, but subsequently its size is decreased compared to the wild type (Yamaki et al., 2011). In the *log-3 fon1-1* double mutant, the FM size is recovered, and the ovules phenotype is largely rescued compared to the *log-3* single mutant. In addition to prevent premature termination of the shoot meristem, *LOG* is also required for ovule formation through maintaining a sufficient volume of the FM to allow normal development of carpels (Yamaki et al., 2011).

In conclusion, studies conducted to date have revealed a close link between plant hormone pathways and carpel establishment in grasses. Although advances have been made in this area, there are still many questions awaiting to be answered, including how the transition between meristem activity and ovule initiation is regulated and whether there are even closer interactions between plant hormone pathways and the C, D, and E class genes relevant to carpel development.

## The Role of miRNAs During Carpel Formation

MiRNAs are small ribonucleic acid molecules (typically 21 nt in length) that negatively regulate gene expression mainly by triggering mRNA cleavage, translational inhibition, or DNA cytosine and/or histone methylation of miRNA target genes (Voinnet, 2009; Liu et al., 2017). Studies conducted so far suggest the role of miRNAs as an important regulatory mechanism in all phases of plant life including flower development (Rubio-Somoza and Weigel, 2011; Xie et al., 2015; Smoczynska and Szweykowska-Kulinska, 2016; Liu et al., 2017).

It seems that miRNA-mediated regulation of floral genes as was observed in *Arabidopsis* also takes place in monocots, such as the suppression of *AP2*-like genes mediated by miRNA172 (Chen, 2004; Chuck et al., 2007; Zhu et al., 2009; Nair et al., 2010). Overexpression of miRNA172 converts sepals and petals into carpels in *Arabidopsis*, a phenotype similar to *ap2* mutants (Chen, 2004). Rice plants overexpressing miR172 phenocopied another *AP2*-like gene mutant named *snb*, displaying multiple changes in flower organ development; the carpel was occasionally replaced by a mosaic organ with a lodicule base and anther apices (Zhu et al., 2009). Defective unfused carpels are also shown in the female flowers of maize *tassel seed4* (*ts4*), a loss-of-function mutant of miRNA172e (Chuck et al., 2007). Mutations in hormonal balance and TFs such as *APETALA2* (*AP2*), *CUP-SHAPED COTYLEDON2* (*CUC2*) and *HECATE* all lead to partially or completely unfused carpels in *Arabidopsis* gynoecium, and *CUC2* has been shown as a target of miR164 (Ripoll et al., 2011; Nahar et al., 2012; Schuster et al., 2015). Using the combination of miRNA sequencing, degradome, and physiological analyses, in total 20 of 162 known miRNAs have been identified to be differentially expressed between incompletely and completely fused carpels in maize (Li et al., 2017). Moreover, 60% target genes of the differentially expressed known miRNAs are found to encode TFs, including those reported to play a role in carpel fusion and development such as auxin response factor (ARF), TB1-CYC-PCFs (TCP), AP2, growth regulating factor (GRF), MYB, and NAC (Li et al., 2017). In barley, the *AP2* ortholog *Cly1* (*cleistogamy1*) is also targeted by miR172; however, the downregulation of *Cly1* seems only to affect lodicule development (Nair et al., 2010). In wheat, mutations to the two *AP2*-like miR172 targets *AP2L5* (also known as gene *Q*) and *AP2L2* resulting in homeotic transformation of lodicule and the adjacent stamen into carpelloid structures (Debernardi et al., 2020). But different from the petal-to-stamen conversion observed in *Arabidopsis ap2* mutants, which is just caused by the expansion of *AtAG* expression in the second whorl, the conversion of lodicules in wheat *ap2l2 ap2l5* mutant is a result of both reduced expression of B-class genes and increased expression of *AG*-like genes (Debernardi et al., 2020).

In *Arabidopsis*, miRNA396 has been characterized to mediate carpel development by suppressing its *GRF* target genes. Pistils with a single carpel are observed in miR396 overexpression plants, these carpel abnormalities can be rescued by miR396-resistant version of GRFs (Liang et al., 2014). GRF interact with its transcription coactivator GRF-INTERACTING FACTOR (GIF) to form the GRF/GIF complex in plant cell nucleus. It is also proposed that the amount of GRF/GIF complex is essential for carpel establishment. While the *gif* single mutant displays normal pistils, the triple mutant *gif1/gif2/gif3* produces abnormal pistils similar to *35S:MIR396a*/*grf5* plants (Liang et al., 2014). Similarly, transgenic rice plants overexpressing miR396 display altered floral organ morphology including abnormal stigma numbers, and this effect is correlated with a significant down-regulation of *GRF6* and other members of this family (Liu et al., 2014). OsGRFs, modulated by their interaction with OsGIF1, directly activate the expression of targets, including *OsJMJ706* and *OsCR4*, which has been reported to participate in the regulation of floral organ development (Sun and Zhou, 2008; Pu et al., 2012; Liu et al., 2014). Overall, increasing evidence suggests a role of microRNAs such as miRNA172 and miRNA396 in carpel development, especially in carpel fusion.

## The Potential Application in Yield Improvement and Hybridization

As productivity depends on grain number and grain weight in many corps (Sreenivasulu and Schnurbusch, 2012; Sakuma and Schnurbusch, 2020), genes and genetic pathways involved in carpel establishment can potentially be manipulated to increase their yield. For example, the increase in carpel number in *fon4* mutants also results in some flower with two seeds that have normal embryos (Chu et al., 2006). Reducing the preprogrammed abortion of floral organs such as pistil primordia abortion in maize tassel spikelets and lateral floret abortion in barley could also be a potential way in yield improvement, as reviewed recently (Gauley and Boden, 2019; Sakuma and Schnurbusch, 2020). However, how to manipulate those genetic components that can improve carpel traits without affecting negatively inflorescence and floral development might be a big challenge. Besides, the completely female-sterile *ospid* mutant lacking stigma and style also shows its potential for applications in hybrid rice production. In traditional hybrid rice production, the two parental lines need to be planted within close proximity, and grains from the two parental lines have to be harvested separately as only grains from the male-sterile maternal line will be hybrid rice, which makes the process labor intensive. On the other hand, in seedling and young adult stages, *ospid* mutants appear quite normal, whereas extra stamens and viable pollen grains are produced in *ospid* flowers (He et al., 2019). Therefore, if sown together with male-sterile lines, an *ospid* mutant might be a good resource for rice hybrid breeding, which will greatly reduce labor and production costs compared with the traditional method, as any grains produced will be hybrid using sterile lines from both parents (He et al., 2019), whereas in this case, a rescue system will be needed for both parents.

## Conclusion and Perspectives

During the last decade, great progress has been made toward understanding developmental mechanisms of carpel establishment. As summarized above, grasses have evolved specific mechanism to regulate carpel identity and stigma formation. However, many conserved regulators are maintained during grass family evolution. TFs in the AG lineage still play essential roles in grass female organ regulation, with conserved functions and conserved interaction partners. The miRNA-mediated carpel regulation pathways are also largely conserved among monocot and dicot plants. Even though some genes and pathways have been discovered in grass carpel development especially in rice and maize, the underlying modes of action are still largely unknown, which should be the future focus. With the increasing availability of gene editing technologies and artificially selected cultivars and the genome-scale expression profiling data, a deeper understanding in cereal crop carpel regulation genes and pathways will be obtained, which will undoubtedly benefit crop breeding programs for grain yield increase.

## Author Contributions

CS, GL, and DZ designed the manuscript. CS, LD, GL, and DZ wrote the manuscript. LD, GL, and DZ critically evaluated the manuscript. All authors contributed to the article and approved the submitted version.

## Conflict of Interest

The authors declare that the research was conducted in the absence of any commercial or financial relationships that could be construed as a potential conflict of interest.
